# Maternal and Birth Characteristics Are Relevant to the Consumption of Ultra-Processed Foods in Young Adults: Results from the Nutritionists’ Health Study

**DOI:** 10.3390/ijerph22091321

**Published:** 2025-08-25

**Authors:** Sthefani C. Penha, Ilana N. Bezerra, Daniela V. Azevedo, Helena A. C. Sampaio, Antonio A. F. Carioca

**Affiliations:** 1 Program Public Health, Faculty of Medicine, Federal University of Ceará, Street Professor Costa Mendes, 1608, Fortaleza 60430-140, CE, Brazil; sthefanicostanutricionista@gmail.com; 2 Nutrition Department, State University of Ceará, Dr. Silas Munguba Avenue, 1700, Fortaleza 60714-903, CE, Brazil; ilana.bezerra@uece.br (I.N.B.); daniela.vasconcelos@uece.br (D.V.A.); 3 Postgraduate Program in Public Health, State University of Ceará, Dr. Silas Munguba Avenue, 1700, Fortaleza 60714-903, CE, Brazil; dr.hard2@gmail.com; 4 Nutrition Department, University of Fortaleza, Washington Soares Avenue, 1321, Fortaleza 60811-905, CE, Brazil

**Keywords:** maternal age, parity, childbirth, maternal exposure, food, processed

## Abstract

Background: One’s dietary pattern throughout life is established during the perinatal period, especially in the intrauterine environment. This study aims to analyze whether maternal and birth characteristics are associated with food consumption in young adults using baseline data from the Nutritionists’ Health Study (NutriHS). Methods: We employed cross-sectional analysis of data from 386 undergraduate nutrition students and nutritionists. Current food consumption was evaluated as per the NOVA classification. The maternal and birth factors included maternal age, parity, type of childbirth, health problems during pregnancy, prematurity, and birth weight, and multiple correspondence analysis of these variables was performed to identify patterns in them. Results: The energy contribution of ultra-processed foods was positively associated with the pattern characterized by participants whose mothers were 19 years of age or younger, primiparous, and had a vaginal delivery (β = 0.48; 95% confidence interval = 0.02, 1.66). Conclusions: We concluded that maternal age at birth was associated with the dietary patterns of adult children. Participants whose mothers were 19 or younger at birth had significantly higher consumption of ultra-processed foods in adulthood compared to those whose mothers were older.

## 1. Introduction

Knowledge of food consumption determinants is essential for understanding how a diet affects health throughout the lifespan [1,2]. Within this context, one’s dietary pattern throughout life is established during the perinatal period, especially in the intrauterine environment [3,4,5]. Thus, studies have confirmed that future health and disease are determined at conception, an event known as metabolic programming [6].

These results are induced by maternal factors, such as maternal age, parity, health problems during pregnancy, and childbirth type, which may be associated with a high-risk pregnancy [7]. Maternal characteristics influence the food consumption of the offspring. To illustrate this, some studies have shown that mothers with adolescent pregnancy, multiparous, and less-educated mothers are most likely to offer unhealthy foods to their children, which can result in an adherence to inappropriate eating habits in adulthood [8,9,10].

Although the relevance of the study of isolated nutrients is recognized, we should consider that there is usually a combined consumption of a variety of foods and nutrients. Noting the importance of evaluating dietary patterns as a complement to the traditional analysis, especially in epidemiological studies, as this allows for significant interventions to be implemented [11]. The NOVA classification is helpful in this investigation because it allows us to evaluate the influence of ultra-processed food on diet and analyze its relationship with health [12].

Currently, there is a limitation of studies involving adults that evaluate the association between maternal and birth events and food consumption in adulthood, especially those using the NOVA classification, because there is a need to conduct longitudinal studies. Therefore, a cohort of undergraduate students studying nutrition and newly graduated nutritionists can be advantageous, since they are, or are in training to become, health professionals who address dietary assessment daily, which makes it possible for us to obtain reliable information. These data are essential for understanding the factors that can influence food consumption throughout the lifespan and prevent associated diseases.

We hypothesized that perinatal events affect food consumption habits during adulthood. In this context, the present study aimed to analyze whether maternal and birth aspects are associated with food consumption in young adults using baseline data from the Nutritionists’ Health Study (NutriHS).

## 2. Materials and Methods

### 2.1. Study Design

This cross-sectional study was conducted with the population participating in the Nutritionists’ Health Study (NutriHS), in Fortaleza, Ceará, Brazil, from 2018 to 2019.

### 2.2. Setting

The Nutritionists’ Health Study (NutriHS) is a longitudinal study with undergraduate nutrition students and nutritionists from three Brazilian universities (the University of São Paulo, in São Paulo, the State University of Campinas, in Limeira, and the University of Fortaleza/State University of Ceará, in Fortaleza), from 2013 to 2022.

### 2.3. Participants

NutriHS Ceará Instagram ads and emails to students and alumni were used to recruit participants across all participating campuses.

Of all the participants recruited, 61% accessed the site and partially completed the self-administered questionnaires. NutriHS sample size estimation required a minimum of 233 volunteers per center, considering the detection of a correlation coefficient of ≥0.20 (α = 5%, β = 20%) and an additional 20% for losses to follow-up [3,13]. The sample size calculation for the present study was based on previously established parameters.

The study included all undergraduate nutrition students and newly graduated dietitians aged 18 or older at baseline. Participants were excluded if they were pregnant at baseline, had incomplete information about the first two years of life, had a deceased mother, were the result of a twin pregnancy, had incomplete food frequency questionnaire (FFQ) data, or had a daily energy intake greater than 5000 kcal. The study totaled 386 participants after exclusions.

### 2.4. Variables

Data on general health were collected through electronic and self-applied questionnaires, including sociodemographic data, current health, early life factors, childhood, current food consumption, and morbid personal and family history [13].

For this study, sociodemographic data (age, sex, skin color, household income, and smoking status) were collected, as well as information on events in the first two years of life, current dietary intake, and current nutritional status. Body mass index (BMI) was calculated from weight (kg) and height (m), obtained through self-reported questionnaires. BMI was categorized as underweight (16–18.4 kg/m^2^), normal weight (18.5–24.9 kg/m^2^), and overweight (≥25 kg/m^2^) [14]. A previous study indicated that self-reported weight and height are accurate measures for calculating BMI in adults [15].

Past data were collected from the application of questions regarding information about the mother and the participant in the first 2 years of life. The participant was instructed to consult his or her mother and birth certificate to obtain reliable data. In particular, the following maternal information was collected: age (≤19 years, 20–34 years, or ≥35 years), education (<11 years or ≥11 years), prenatal care (yes or no), number of prenatal care visits (<6 consultations or ≥6 consultations), parity (first pregnancy, second pregnancy, or third or higher pregnancy), health problems during pregnancy (yes or no), and childbirth type (natural or cesarean). Regarding the participant’s data at birth, information on prematurity (yes or no) and birth weight (<2.5 kg, 2.5–3.9 kg, or ≥4.0 kg) was collected. Birth weight was classified as low weight (<2500 g), adequate weight (3000–3999 g), and macrosomia (≥4000 g) [16].

Current food consumption was assessed through the application of a previously validated quantitative food frequency questionnaire (FFQ) [17]. The quantitative FFQ assessed the eating habits of individuals over 1 year, identifying the usual individual portion of 136 food items, with questions on frequency options (0 to 10 times), time units (day, week, month, or year), and portions (described using household measurements, grams, or millimeters). Food consumption was evaluated as per the NOVA classification, which categorizes foods into four groups: in natura or minimally processed foods, processed culinary ingredients, processed foods, and ultra-processed foods [12].

From the FFQ, the food items were classified into these groups, with 74 food items in the in natura or minimally processed food group, 3 in the processed culinary ingredient group, 19 in the processed food group, and 40 in the ultra-processed food group. This classification is similar to that adopted in a previous study [3].

From this classification, the percentages of the contribution of each group to the daily energy intake were calculated based on the method used in a previous study [18]. Thus, the amount of the portion reported in the FFQ was transformed into an equivalent amount of a household measurement (grams or milliliters). The reported consumption frequency was converted into a daily frequency and then associated with the reported portion to calculate the energy value of each food item (primary outcome variable). For this calculation, the information obtained from the food composition table of the United States Department of Agriculture (USDA) was considered [19,20].

### 2.5. Data Sources

The questionnaires were presented in blocks of questions, using the e-NutriHS system (http://www.fsp.usp.br/nutrihs, accessed on 18 May 2022).

### 2.6. Bias

We adopted methodological strategies, such as statistical analyses adjusted for potential confounding variables and the comparison of results with longitudinal evidence available in the literature in order to minimize the risk of bias.

### 2.7. Statistical Methods

**Figure 1:**
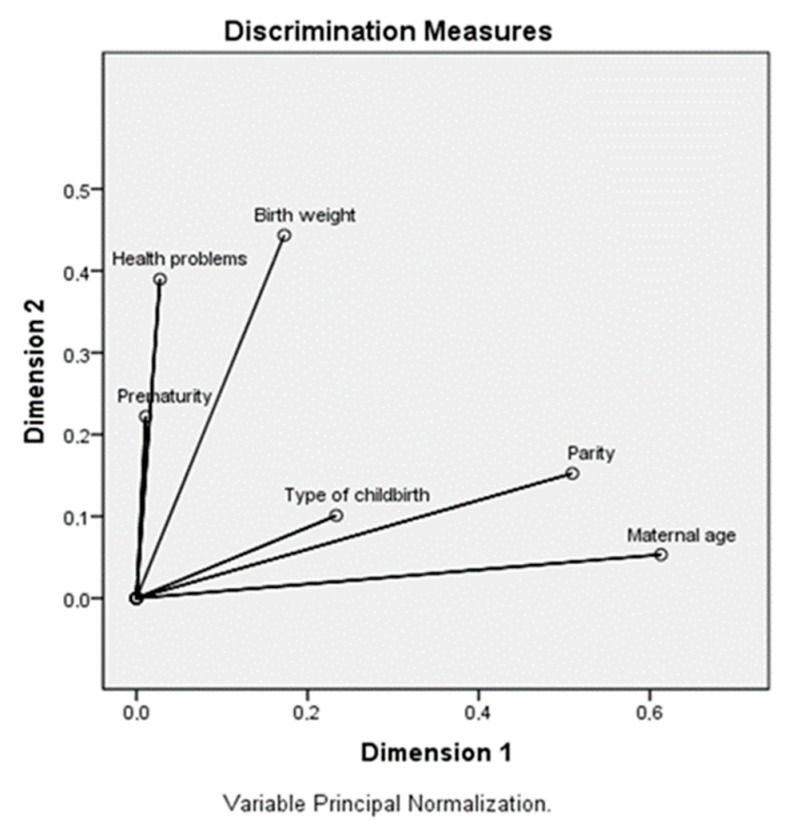
Spatial representation of relationships between the derived factors and maternal characteristics.

The description of the variables was expressed in absolute and relative values or mean and standard deviation. Multiple correspondence analysis with maternal and birth variables (maternal age, parity, type of childbirth, health problems during pregnancy, prematurity, and birth weight) was used to derive two patterns (Figure 1). Pattern adherence scores were obtained for each volunteer. Multiple correspondence analysis is defined as a descriptive and exploratory statistical analysis to verify the simultaneous relationships between two or more categorical variables, forming groups [21].

We employed the analysis of variance to compare the mean scores between the predictor variables of the multiple correspondence analysis. A multiple linear regression model was performed to identify the association between adherence scores to patterns of maternal and birth aspects (independent variable) and current energy consumption of food groups per the NOVA classification (dependent variable). The beta estimation and 95% confidence interval (95% CI) were calculated in the crude model and adjusted for sex (male or female), age (years), household income (<1, 1–5, 6–10, or >10 Brazilian minimum wages), and BMI (kg/m^2^). The selection of covariates was based on known confounding factors reported in the literature (sex, age, household income, and body mass index) [3,4]. Statistical analyses were performed using Stata software (version 12.0, 2011, StataCorp LP, College Station, TX, USA). *p*-value < 0.05 was used to indicate statistically significant differences.

## 3. Results

Approximately 82.6% of the 386 total participants (mean age, 23.9 ± 6 years) were female, 55.2% self-declared as Black/Brown, 39.4% reported that the head of the family had completed high school, and 59.3% had a household income of 1 to 5 minimum wages. The mean BMI was 24.2 ± 4.1 kg/m^2^, and 60.1% were classified as eutrophic. The current mean daily energy intake was 2565 ± 896 kcal, 62.2% of which derived from in natura and minimally processed foods, 3.3% from processed culinary ingredients, 19.3% from processed foods, and 15.1% from ultra-processed foods (Table 1).

Regarding the events that occurred in the first 2 years of life, there was a predominance of mothers aged 20–34 (67.1%), 47.9% were primiparous, 13.7% had health problems during pregnancy, and 51.8% underwent cesarean childbirth. For the participants, 6.7% were born prematurely, and 13.5% were born with an inadequate weight (low birth weight and macrosomia) (Table 2).

Two patterns were identified among the maternal and gestational variables. The first pattern, Pattern 1, was associated with a maternal age of 19 years or younger, parity of the first pregnancy, and a natural childbirth (*p*-value < 0.001). The second pattern, Pattern 2, was characterized by health problems during pregnancy, prematurity, and an inadequate birth weight (low birth weight and macrosomia) (*p*-value < 0.001) (Table 3).

After adjusting for confounders, the current energy contribution of ultra-processed foods was positively associated with Pattern 1, which was characterized by participants whose mothers were 19 years old or younger, were primiparous, and had a natural childbirth (β = 0.84; 95% confidence interval—95% CI = 0.02, 1.66). There was no association between the energy contribution of foods per the NOVA classification in adulthood and Pattern 2 (Table 4).

## 4. Discussion

This is the first study to assess the association between maternal and birth factors and food consumption in adulthood using the NOVA classification, which considers food processing levels. We observed that the group of mothers with adolescent pregnancy, made up of those participants whose mothers were 19 years of age or younger, were primiparous, had a natural childbirth, and had the highest consumption of ultra-processed foods in adulthood.

We observed that 34.4% of current food consumption derived from processed foods and ultra-processed foods, and 15.1% of this was attributed solely to the intake of ultra-processed foods. In Brazil, the percent intake of ultra-processed foods is 14.8% according to the Family Budget Survey (FBS) 2017–2018 [22]. By comparing our results with nationally representative data, we observed a consumption similar to that of the Brazilian population. However, due to the knowledge of the effects of nutrition on health in the patients of our sample, which was composed of nutritionists in training and graduated nutritionists in the workforce, we expected that the observed food consumption would be more consistent with that in the recommendations of the Food Guide for the Brazilian Population [23].

Our results show that participants whose mothers were 19 years old or younger at birth had significantly higher consumption of ultra-processed foods in adulthood compared to those whose mothers were older. This association is in line with the findings of the Etiology of Preterm Birth and Consequences of Perinatal Factors for Child Health cohort study, which reported that children of adolescent pregnancy had greater adherence to unhealthy dietary patterns [8]. Although our analysis did not adjust for all potential confounders, such as maternal dietary, this result may reflect the influence of maternal experiences on early-life feeding practices and long-term eating behaviors. Adolescent mothers likely have the least access to information about appropriate eating habits and the greatest access to and preference for processed foods.

Primiparity was significantly associated with the consumption of ultra-processed foods by participants in adulthood, coinciding with the fact that they were children of mothers with adolescent pregnancy, and primiparity is more common in younger women. However, this finding contrasts with the results in the existing literature, which implies that primiparity is associated with a lower intake of processed foods by the offspring, such as ready-to-eat dairy products, cookies, confectioneries, and sugary drinks [10]. Our study’s findings could be explained by the lower level of knowledge and experience of primiparous mothers with infant feeding, which influences eating habits in adulthood.

Faced with this reality, there is a need to improve access to information and actions that promote healthy eating practices beginning at early childhood, and this should start during pregnancy through prenatal care visits. In Brazil, Rede Alyne aims to guarantee humanized care for the mother-child binomial, and among the types of care encouraged are family planning, the promotion of breastfeeding from prenatal care, and the introduction of adequate complementary foods for children from the age of 6 months [24].

Through the document “Third Issue—Protocol for the Use of the Food Guide for the Brazilian Population in the Nutritional Guidance of Pregnant Women”, the Ministry of Health provides dietary guidelines for pregnant women based on the Food Guide for the Brazilian Population. These are essential for adequate maternal health and nutritional status [25]. The World Health Organization recommends prioritizing the consumption of a variety of foods by pregnant women, including fruits, green and orange vegetables, milk, beef, poultry, fish, beans, nuts, and whole grains, as well as the use of supplements, such as folic acid and iron, and certification of food origin [26].

Additionally, the Amamenta e Alimenta Brazil Strategy was created to promote breastfeeding and healthy complementary feeding for children younger than 2 years through the qualification of professionals in primary health care regarding the theme [27]. In this context, these professionals, who are mainly nutritionists, should encourage and disseminate information about the importance of adequate nutrition in this stage of life based on the Food Guide for Brazilian Children Under 2 Years, as this will help develop healthy eating habits and the prevention of chronic diseases in adult life [28].

We demonstrated that natural childbirth was related to a higher intake of ultra-processed foods in adulthood. The literature implies that natural childbirth is common among women younger and primiparous mothers [29], and, as there was a high prevalence of these mothers in our study, being a mother with adolescent pregnancy may be correlated with having a vaginal childbirth, influencing the greater participation of ultra-processed foods in the current diet of the offspring.

Confounding variables may have also influenced this result because, although they were adjusted for, other variables may be associated with natural childbirth and the consumption of ultra-processed foods. For example, a study conducted in Brazilian mothers and babies aged between 6 and 24 months identified that the introduction of ultra-processed foods in the complementary feeding of a child results from an increased maternal consumption of these food products and a lack of breastfeeding [30]. A cross-sectional analysis conducted in young adults revealed that, when they were fed infant formula early on (before 1 month of age), they were prone to the highest consumption of ultra-processed foods in young adulthood [3].

Our results have limitations that must be considered. As prior information was worked on in the study, this may imply that memory bias exists. However, previous searches have shown that these variables are reported accurately for up to 20 years after birth [31]. As the mean age of our participants was similar, the interference of this type of bias in our results could be attenuated. The study cohort is made up of undergraduates from the nutrition course and graduated nutritionists, characterized by homogeneity, and this may not allow for the results to be generalized to other populations, interfering with external validity. However, it is a strong aspect of our study, because they are aware participants of the importance of providing reliable information on food intake and are more familiar with the tools used for dietary assessment, ensuring internal validity.

The availability and cost of food, the local culture, and the study/work routine are also noteworthy determinants of adherence to this habit. Currently, ready-to-eat foods are increasingly used as substitutes for in natura or minimally processed foods, as well as for meals prepared in the home environment [32], among younger people, pregnant or non-pregnant women, and men [33] as a result of accelerated urbanization. Age has been shown to have an important effect on eating behavior. Younger individuals tend to adopt inadequate eating habits, such as replacing main meals with snacks and drinking energy-dense drinks, as a result of greater susceptibility to commercial marketing [34]. In quantitative analyses, it is difficult to control these types of confounders, as was in the present study, and this may lead to an association between vaginal childbirth and higher current consumption of ultra-processed foods despite implementing statistical controls for measurable confounders.

## 5. Conclusions

We concluded that maternal age at birth was associated with the dietary patterns of adult children. Participants whose mothers were 19 or younger at birth had significantly higher consumption of ultra-processed foods in adulthood compared to those whose mothers were older. This finding supports the hypothesis that perinatal factors, such as teenage pregnancy, can influence long-term dietary behaviors, potentially through early exposure to family dietary practices. Our results highlight the importance of considering maternal experiences in nutritional counseling and preventive strategies throughout life. Prospective studies with larger sample sizes and of different populations are essential to confirm the results observed in this study and to elucidate inconsistencies in the literature on the association between maternal and birth factors and food consumption habits in adulthood.

## Figures and Tables

**Table 1:** Sociodemographic characteristics, nutritional status, physical activity level, and food consumption per the NOVA classification of young adults from the NutriHS study in Fortaleza, Brazil.

Variables	n	%
**Sex**		
Female	319	82.6
Male	67	17.4
Age, in years ^a^	23.9	6.0
**Self-reported skin color**		
White	159	41.2
Black/Brown	213	55.2
Others	14	3.6
**Family head schooling**		
Never attended school/Incomplete Elementary School (from 1 to 7 schooling years)	82	21.3
Complete Primary Education (from 8 to 10 schooling years)	34	8.8
Complete High School (from 11 to 13 schooling years)	152	39.4
University (14 years or more of study)/Postgraduate	118	30.5
**Household income**		
<1 Minimum Wage	47	12.2
1–5 Minimum Wages	223	59.3
6–10 Minimum Wages	49	12.7
>10 Minimum Wages	29	7.5
**BMI, kg/m^2 a^**	24.2	4.1
**Moderate and intense physical activity level**		
<150 min/week	111	28.8
>150 min/week	275	71.2
**NOVA** **classification ^b^**		
Unprocessed and minimally processed, % total energy	62.2	13.5
Culinary ingredient, % total energy	3.3	3.5
Processed, % total energy	19.3	11.8
Ultra-processed, % total energy	15.1	8.5

^a^ Value expressed as mean and standard deviation. ^b^ Percentage values that do not result in 100% refer to the missing.

**Table 2:** Maternal age, education, prenatal care, parity, pregnancy complications, delivery type, prematurity, and birth weight of the mothers of young adults from the NutriHS study in Fortaleza, Brazil.

Maternal age	n	%
≤19 years	52	13.5
20–34 years	259	67.1
≥35 years	47	12.2
**Maternal** **schooling**		
<11 years	146	37.8
≥11 years	234	60.6
**Prenatal care visits**		
Yes	231	93.1
No	17	6.9
**Number of prenatal** **care visits**		
<6 visits	40	16.9
≥6 visits	197	83.1
**Parity**		
1st gestation	185	47.9
2nd gestation	117	30.3
≥3rd gestation	79	20.5
**Health problems during pregnancy, yes**	53	13.7
Arterial hypertension	18	3.3
**Childbirth type**		
Natural	181	46.9
Cesarean Section	200	51.8
**Prematurity**		
No	355	92.0
Yes	26	6.7
**Birth weight**		
<2.5 kg	24	6.2
2.5–3.9 kg	313	81.1
≥4.0 kg	28	7.3

**Table 3:** Differences in adherence scores to maternal and gestational patterns by maternal age, parity, delivery type, pregnancy complications, prematurity, and birth weight.

Pattern 1		
**Maternal age**	Mean	SE
≤19 years	1.16	0.07
20–34 years	0.10	0.04
≥35 years	−1.87	0.09
*p*-value	<0.001	
**Parity**		
1st gestation	0.59	0.06
2nd gestation	0.02	0.06
≥3rd gestation	−1.30	0.09
*p*-value	<0.001	
**Childbirth type**		
Natural	0.52	0.06
Cesarean Section	−0.45	0.07
*p*-value	<0.001	
**Pattern 2**		
**Health problems during pregnancy**		
No	−0.27	0.04
Yes	1.54	0.15
*p*-value	<0.001	
**Prematurity**		
No	−0.13	0.87
Yes	1.75	1.24
*p*-value	<0.001	
**Birth weight**		
<2.5 kg	2.22	0.19
2.5–3.9 kg	−0.25	0.04
≥4.0 kg	1.13	0.19
*p*-value	<0.001	

Values expressed as mean and standard error. Comparison of means by ANOVA test. Percentage values that do not result in 100% refer to the missing. Pattern 1 was associated with a maternal age of 19 years or younger, parity of the first pregnancy, and natural childbirth, while Pattern 2 was characterized by health problems during pregnancy, prematurity, and inadequate birth weight, including both low birth weight and macrosomia.

**Table 4:** Association between maternal and birth characteristics and current energy consumption in young adults from the NutriHS cohort in Fortaleza, Brazil.

NOVA Classification	Pattern 1		Pattern 2	
	Crude Model	Adjusted Model *	Crude Model	Adjusted Model *
	Beta	95% CI	Beta	95% CI
Unprocessed and minimally processed, % total energy	−0.95 (−2.26; 0.36)	−0.90 (−2.19; 0.40)	0.23 (−1.10; 1.56)	0.26 (−1.10; 1.61)
Culinary ingredient, % total energy	0.00 (−0.35; 0.34)	0.01 (−0.33; 0.35)	−0.35 (−0.69; 0.01)	−0.25 (−0.61; 0.10)
Processed, % total energy	0.07 (−1.07; 1.21)	0.05 (−1.09; 1.18)	−0.253 (−1.41; 0.90)	−0.22 (−1.40; 0.97)
Ultra-processed, % total energy	**0.88 (0.06; 1.70)**	**0.84 (0.02; 1.66)**	0.37 (−0.47; 1.21)	0.22 (−0.64; 1.07)

* Model adjusted for sex, age, household income, and body mass index. In bold, beta values with *p*-value < 0.05. Confidence interval (95% CI). Pattern 1 was associated with a maternal age of 19 years or younger, parity of the first pregnancy, and natural childbirth (*p*-value < 0.001), while Pattern 2 was characterized by health problems during pregnancy, prematurity, and inadequate birth weight, including both low birth weight and macrosomia.

## Data Availability

Due to ethical and legal restrictions, the data underlying this study are available upon request. The Research Ethics Committee of the University of Fortaleza, which imposed the ethical restrictions, can be contacted by email or telephone. To request access to this data, interested researchers must submit a detailed project proposal to the aforementioned Ethics Committee via email (coetica@unifor.br) or by telephone (+55-8534773122). The authors confirm that the data underlying this study will be shared as long as requests are submitted through the appropriate channels. Augusto Carioca, author of the current study, can be contacted to help facilitate data access requests via email (carioca@unifor.br).
